# Supplementary material for: Pulmonary acini exhibit complex changes during postnatal rat lung development
Source: PLoS One. 2021 Nov 8;16(11):e0257349. doi: 10.1371/journal.pone.0257349 (PMC8575188; doi:10.1371/journal.pone.0257349)
Supplement: Attachment — Submitted filename: RebuttalLetter.pdf [file pone.0257349.s001.pdf]

b
UNIVERSITÄT
BERN

Faculty of Medicine
Institute of Anatomy

Institute of Anatomy, Baltzerstrasse 2, CH-3012 Bern

PLOS ONE
Josué Sznitman, Academic Editor
Two anonymous reviewers

Bern, July 16, 2021

Rebuttal letter for *PONE-D-21-11732*: Pulmonary acini exhibit complex changes during postnatal rat lung development

Dear Josué

We thank you for the opportunity to submit a rebuttal letter for our manuscript on the size distribution of rat lung acini (*PONE-D-21-11732*). Below you will find our 'point-by-point' response to the issues mentioned by you and the comments of the two reviewers for your consideration.

To the Editor:

> In particular, the aim of the manuscript could be stated more specifically, instead of expressing it in general terms, so that readers can know the importance of the work.

We reformulated the aims subsection in the introduction and defined our aims more precisely and thank the editor and reviewers for the input to improve this section.

"Furthermore, citations are not fully appropriate and should be improved prior to publication."

If by *not fully appropriate* you mean *lacking*, then we have (happily) added the reference that one reviewer suggested. If by *not fully appropriate* you mean *not formatted well*, then excuse ourselves until the manuscript is accepted. We prepared the manuscript with manubot and used the PLOS ONE CSL style for generating to originally submitted .DOCX file. The final version of our manuscript passing the peer-review will make use of the PLOS ONE LaTeX template, which then should take care of all formatting issues.

> Please ensure that your manuscript meets PLOS ONE's style requirements, including those for file naming. The PLOS ONE style templates can be found at [. . .]

See above.

David Haberthür
Post-Doc
Baltzerastrasse 2
CH-3012 Bern

Phone +41 31 634 50 74
haberthuer@ana.unibe.ch
anatomie.unibe.ch/haberthu

> Please remove your figures from within your manuscript file, leaving only the individual TIFF/EPS image files, uploaded separately.

See above.

To Reviewer #1:

> [. . .]I have the following criticism and comments on their stated aims [. . .] While I generally agree with the authors' stated aim, I wonder how the knowledge of detailed acinar structure—opposite of global knowledge—would change the predictions of the current computational fluid dynamics simulations of airflow qualitatively. I am not convinced that CFD simulations based on the current new data would yield new knowledge beyond what we already know experimentally [see ref. 15].

Computational fluid dynamic simulations are usually based on highly idealized and simplified acinar geometries. As we found the shape of the alveoli to be rather irregular, we think that the irregular shape and volume distribution of the acini could substantially influence the CFD simulations. We updated the paragraph and tried to explain this in a more detailed manner.

> In the second paragraph of the "Aims of this study", the authors mentioned the effects of individual variations of acinar volumes during the postnatal lung development on CFD.

We do understand the argument of the reviewer. She/He is right that the global view of acinar particle deposition is well characterized by the simulations. However, the shape of the alveoli is rather irregular. Therefore, looking to the precise location of particle deposition inside individual alveoli, we expect that the shape of the alveoli may have an influence. Furthermore, in (partly unpublished) multi-breath nitrogen washout simulations (Hasler et al. [@doi:10.1371/journal.pcbi.1007079]), we were able to show that the range and the compliance of the acinar size has an influence on the time needed for the gas washout, or ventilation of the acini, respectively. We updated the paragraph accordingly.

> I agree with the authors that changes in the individual variation of acinar volumes would likely to change acinar fluid mechanics. It would be important, however, to illustrate the potential effect of the observed new data (i.e., a reduction in variation by a factor of 6-7) on change in fluid mechanics by briefly summarizing Ref. 13 and perhaps the authors' unpublished results.

We added a short summary of the reference in question at the end of the 'aims' section

> Please give us a rational that a voxel size of 1.48mm is sufficient to achieve the objective of the current study.

We imaged the lungs with a voxel size of 1.48 μm and not 1.48 mm. We suspect this is a typo from the reviewer.

The voxel size of 1.48 μm we used to image our samples represents a compromise between the resulting field of view and the limited scanning time available at the used synchrotron beamline. In (unpublished) preliminary studies we did not encounter differences in the segmentation of the alveolar septa and the acini when comparing a voxel size of 0.75 μm and 1.48 μm. The larger voxel size of 1.48 μm we used roughly cut the scanning time half, and is sufficient to resolve the alveolar septa. In the study of Barré et al. [@doi:10.14814/phy2.12063] we determined the required voxel size for the detection of acini to be 3.6 μm, which is more than twice of the voxel size we used in this study.

> To make the references more appropriate, the authors should mention *Das GK [. . .] Novel multi-functional europium-doped gadolinium oxide nanoparticle aerosols facilitate the study of deposition in the developing rat lung [. . .]* together with ref. 16 and they should mention that both of these publications agree with the original results of Ref. 15.

Das et al. [@doi:10.1039/c6nr00897f] looked at the particle deposition both normalized to body weight and mass of lung tissue. In the abstract they mention that “Neonatal, juvenile and adult rats (1, 3 and 12 weeks old) were [. . .] exposed to [. . .] nanoparticles [. . .]. The dose of deposited particles was significantly greater in the juvenile rats [. . .] compared to [. . .] the adult and neonate rats, respectively.” This corresponds well to what we mention in the discussion. We thank the reviewer for bringing this work to our attention and thankfully added the manuscript to our list of references.

To Reviewer #2:

> The authors described the text repeatedly especially for results and discussion part. The author could short the paper in a more concise way.

We duly noted the suggestion from the reviewer, but refrained from making large alterations to the general structure of the text. We are aware that we repeated text between the results and discussion sections of the manuscript, but think it is necessary for readers that might only skim one of the two sections. We feel the structure of the manuscript is acceptable like this but are open to further suggestions.

> A better single acinus image is to be expected in Figure 1 as it is not intuitively clear how the authors obtained acinus delineation. For example, show a high resolution acinar image with full boundaries.

The process of acinus delineation is described in detail in our older publications on the method [@doi: 10.1152/japplphysiol.00642.2013; @doi: 10.1152/ajplung.00325.2016], which are also cited in the text. We thus did not explain the method in much detail. But we fully agree with the suggestion to add 3D visualizations to figure 1 and updated the figure with an additional row showing visualizations of exemplary acini.

> Why the authors displayed the figures in both e.g., linear scale and log. scale for the same results? They could consider using a scale “break” in y-axis to better illuminate

> their results.

We aimed to provide the reader of our manuscript the full picture of the data and thus showed both the linear and logarithmic scale of the data points in their full extent. We don't think that a scale break is a sensible choice to completely show large differences in y-values. In addition, human senses are logarithmic (see en.wikipedia.org/wiki/Weber–Fechner_law), making a presentation of the data in logarithmic scale both pleasing and easier to grasp.

We are happy to update our graphs with only one version of the plots if the reviewer deems it necessary. But we kindly refuse to plot them with a break in the y-axis, which can mislead the reader (see en.wikipedia.org/wiki/Misleading_graph#Truncated_graph)

> The authors described the statistical P value with "better" many times, for example, figure 2, "all p values better than 1.9e-5, which is the one between days4 and 10". Guess here the author meant "better" refers to "larger". The latter (or other more specifically description) is more scientifically sound.

We used *better* in the sense of smaller, i.e. more significant p-values. As stated in the second-to-last paragraph of the Materials & Methods section, we specifically want to avoid giving binary p-values (i.e. p¡0.05), as suggested by Amrhein et al. [@doi:10.1038/d41586-019-00857-9]. We clarified the wording by replacing the relevant instances of *better* with *smaller than* throughout the manuscript.

"One of the important findings here is the decreased numbers of acini from day 4 to day 60."

As we write in the text, we found—within our margin of error—no difference in the number of acini between days 4 and 60. The standard deviation of our results at day 4 is very high, because of the results of sample 04A. As the number of acini was technically a byproduct of our analysis, we *can* calculate it from our data, but the study was not set out to analyze this, we only use this comparison in the 'discussion' section.

> However, the results from the authors' early study demonstrated a constant acinar number in rats, despite using a different strain of rats. The difference is so obvious and why this variation presented in old and current studies. Which result is more scientifically or physiologically correct and why?

The older study from Barré et al. is much more accurate in relation to *this* result, because the underlying question of the number of acini was answered with direct measurement. In our study we can estimate the number of acini based on the assessed volumes of the acini. Due to error propagation, these results in our study are less stringent than the numbers of Barré et al., which we discussed in detail.

"Figure 6, 7, and 8 could be mentioned/placed in the results section rather than discussion part."

We thank the reviewer for this suggestion but feel that these figures are only relating our results to other result in the literature, and hence left them where they are in the discussion section of our manuscript. If the 'could' is actually more a 'should', we are happy to incorporate the changes, though.

> Checking out the misspellings and typos. Page 23, last paragraph. “All the studies mentioned my Osmanagic et al. were performed. . . .”. “my” should be corrected to “by”? Page 25, the last sixth line, “. . . acinar similar sizes effects gas-washout”. Here affects?

We thank the reviewer for detecting these typos and notifying us. Those typos (and some more) have been corrected.

> Page 24, last line. “our values day 4: $4.98 \times 10^5\,\mu m^3$, day 10:. . . ”. “our values” is confusing here because the authors mentioned our values early with different numbers.

In the last section of the results we mention these values the only time before this instance. As far as we see this, the numbers are exactly the same. We kindly ask the reviewer to help us pinpoint the issue if it still persists in the manuscript. We are very happy to iron out any remaining uncertainties in our manuscript.

> Discussion part, it is not clear that why the larger variation of acini size increases the gas-washout time.

The statement is based on partly unpublished results. First, it is shown by a simulation of gas-washout. Second, large acini need a longer time for gas-washout. It seems that the faster washout of the smaller acini is not able to compensate for the slower washout of the larger acini.

Based on a comment of Reviewer#1 we expanded the ’aims’ section with a short explanation regarding this issue.

We thank the reviewers for their work to improve our manuscript and are looking forward to their further comments.

Yours sincerely

David Haberthür
